# MetaComp: comprehensive analysis software for comparative meta-omics including comparative metagenomics

**DOI:** 10.1186/s12859-017-1849-8

**Published:** 2017-10-02

**Authors:** Peng Zhai, Longshu Yang, Xiao Guo, Zhe Wang, Jiangtao Guo, Xiaoqi Wang, Huaiqiu Zhu

**Affiliations:** 1 State Key Laboratory for Turbulence and Complex Systems, Department of Biomedical Engineering, College of Engineering, Peking University, Beijing, 100871 China; 2 Center for Quantitative Biology, Peking University, Beijing, 100871 China; 3 Center for Protein Science, Peking University, Beijing, 100871 China

**Keywords:** Comparative metagenomics, Comparative meta-omics, Statistical analysis, Visualization, Graphical user interface

## Abstract

**Background:**

During the past decade, the development of high throughput nucleic sequencing and mass spectrometry analysis techniques have enabled the characterization of microbial communities through metagenomics, metatranscriptomics, metaproteomics and metabolomics data. To reveal the diversity of microbial communities and interactions between living conditions and microbes, it is necessary to introduce comparative analysis based upon integration of all four types of data mentioned above. Comparative meta-omics, especially comparative metageomics, has been established as a routine process to highlight the significant differences in taxon composition and functional gene abundance among microbiota samples. Meanwhile, biologists are increasingly concerning about the correlations between meta-omics features and environmental factors, which may further decipher the adaptation strategy of a microbial community.

**Results:**

We developed a graphical comprehensive analysis software named MetaComp comprising a series of statistical analysis approaches with visualized results for metagenomics and other meta-omics data comparison. This software is capable to read files generated by a variety of upstream programs. After data loading, analyses such as multivariate statistics, hypothesis testing of two-sample, multi-sample as well as two-group sample and a novel function—regression analysis of environmental factors are offered. Here, regression analysis regards meta-omic features as independent variable and environmental factors as dependent variables. Moreover, MetaComp is capable to automatically choose an appropriate two-group sample test based upon the traits of input abundance profiles. We further evaluate the performance of its choice, and exhibit applications for metagenomics, metaproteomics and metabolomics samples.

**Conclusion:**

MetaComp, an integrative software capable for applying to all meta-omics data, originally distills the influence of living environment on microbial community by regression analysis. Moreover, since the automatically chosen two-group sample test is verified to be outperformed, MetaComp is friendly to users without adequate statistical training. These improvements are aiming to overcome the new challenges under big data era for all meta-omics data. MetaComp is available at: http://cqb.pku.edu.cn/ZhuLab/MetaComp/ and https://github.com/pzhaipku/MetaComp/.

**Electronic supplementary material:**

The online version of this article (doi:10.1186/s12859-017-1849-8) contains supplementary material, which is available to authorized users.

## Background

High-throughput meta-omic approaches over the last few years have facilitated researches on understanding of the unculturable majority of microorganisms on earth. Environmental and clinical microbiota samples are characterized in metagenomics, metatranscriptomics, metaproteomics and metabolomics levels. Metagenome reveals taxonomic composition and functional genes abundance. Metatranscriptome accompany with metaproteome further reflect the temporal fluctuation of gene expression. Metabolome identifies metabolites associated with phenotype and physiology as biomarkers. Previously, biologists focused on one or part of all types of meta-omic information, while the integration of metagenomics, metatranscriptomics, metaproteomics and metabolomics data has begun to gain attention for the purpose of systematically characterizing complex microbial communities [1]. Therefore, related bioinformatics tools for processing all types of meta-omics data is in urgent need.

Though the combination of meta-omics approaches may describe a single microbiota in a systems-level, the functional genomic traits associated to host niches and ecological habitats remains obscure. Therefore, it is necessary to introduce comparative meta-omic methods, which refers to statistically comparing meta-omics data from two or more microbiota samples. During the past decades, comparative meta-omics analysis has been established as a routine procedure applied in human pathology and ecology studies. Researchers have already discovered host-specific genes in human gut microbiotas from comparisons between obese and lean volunteers [2], between long- and short-term dietary volunteers [3, 4] and between patients of nonalcoholic fatty liver disease (NAFLD) [5] or irritable bowel syndrome (IBS) [6] and healthy control volunteers. Meanwhile, by applying these techniques, many studies have reported that the composition of microbial community varies with depth of ocean [7, 8] and oscillates seasonally in Western English Channel [9]. Gene expression pattern of a microbiota fluctuates during different growth stages in Acid Mine Drainage (AMD) [10]. Furthermore, it is notable that an increasing number of studies pay attention on measuring physiological or ecological variables for comprehensively investigating the responds of microbial communities to environmental factor variations [3, 4, 7, 11, 12]. This trend requires bioinformatics tools not only to distinguish environmental effects on microbiotas through *p*-value from hypothesis testing or correlation analysis but also to unveil intrinsic mechanisms by statistical modeling such as regression analysis.

For comparative metagenomics, the first tool named as XIPE-TOTEC offered two-sample test and utilized metagenomic shotgun sequences as input [13]. Then, MEGAN was designed to perform barplot for comparing multiple samples clustered in taxonomic or functional clustering views and integrate all types of meta-omics data except metabolomics data in the latest version [14]. IMG/M is a web portal supporting a systematical service containing taxonomic classification, sequence assembly, functional annotation and differential abundance analysis for two- and multi-sample comparison of metagenomic reads [15]. Another comparative metagenomics analysis tool, STAMP, mainly exploits Fishers’s exact test in two-sample test and *t*-test in two-group samples. [16]. MetaStats, developed for two- and multi-sample comparison was exploited on data normalization for metagenomic data [17]. Later on, FANTOM emphasized its ability in comparison between two groups of metagenomic samples which was implemented with user-friendly graphical interface [18].

Several bioinformatic programs had been developed for comparative metagenomics, however few tools were specialized for metatranscriptomics, metaproteomics and metabolomics data comparison (see Table 1 for details). To compare metatranscriptomics samples, metagenomeSeq were often introduced in 16S rRNA, marker-gene expression, RNA-seq data abundance comparison. It was capable for correcting bias caused by variations on sequencing coverage [19]. As for metaproteomics data, MEGAN and STAMP were reported able to process. While only XCMS, an online metabolomic processing platform, performs two-group comparison [20].

**Table 1:** Input data for available comparative meta-omic tools

Tool	Meta-omics	Input Format	Hypothesis	Multiple Testing	Reference
	Data^a^		Testing Modes^b^	Correction^c^	
XIPE-TOTEC	GS	SEED output, APM format.	TST	Bonferroni and FDR	[13]
MEGAN6	GM, GR, GS,	BLAST, RDP classifier, SIN	NA	NA	[14]
	TM*, TS*	-A and STAMP outputs; AP			
	and P	-M in CSV format, BIOM,			
		DAA and SAM files.			
IMG/M4	GR, and GS	MG-RAST and BLAST out	MST and TST	NA	[15]
		-puts; APM, BIOM, fasta			
		and fastq files.			
STAMP	GM, GR, GS,	MG-RAST, IMG/M, CoMet	MST, TGST and	Bonferroni, FDR and	[16]
	TM*, TS*	and RITA outputs; APM a	TST	Šidák	
	and P*	-nd BIOM files.			
Metastats and	GM, GR, GS,	APM and BIOM files.	MST and TST	Bonferroni and FDR	[17, 19]
metagenomeSeq	TM and TS				
Fantom	GR, GS	CAMERA, MG-RAST and	TGST and TST	Bonferroni and FDR	[18]
		IMG/M outputs.			
XCMS	B	cdf, mzData, mzData.XML,	MST and TGST	FDR	[20]
		mzXML, netCDF, wiff			
		and wiff.scan			
MetaComp	GM, GR, GS,	BLAST, HMMscan, IMG/M,	MST, TGST and	Bonferroni and FDR	This work
	TM, TS, P	MG-RAST, MZmine, Kraken	TST		
	and B	and PhymmBL outputs; APM			
		and BIOM files.			

^a^Asterisk (*) denotes that the data types are not designed to be processed but compatible with this tool as an input. Abbreviation of meta-omics data types: GM: amplicon sequenced metagenomic marker gene sequeneces; GR: amplicon sequenced 16S rRNA sequences; GS: shotgun sequenced metagenomic sequences; TM: amplicon sequenced metatranscriptomic marker gene sequences; TS: shotgun sequenced metatranscriptomic sequences; P: metaproteomic sequences. B: metabolomic data

^b^Abbreviation of hypothesis testing modes. MST: multi-sample test; TGST: two-group sample test; TST: two-sample test

^c^FDR denotes for false discovery rate correction

Recently, the rapid accumulation of all types of meta-omics data brings out three major challenges. Firstly, most comparative analysis tools focused on one type of meta-omics data. A universal analysis tool, which is applicable for all types of meta-omics data, will be convenient for researchers characterizing microbiota in multiple meta-omics levels. Secondly, all these tools paid no attention to unveil the correlation between microbiota and its living conditions such as temperature, humidity, pH value and salinity. Lacking of this analysis will definitely hamper biologists from deciphering the microbial adaptive strategy and other interaction between microbes and habitats. Finally, as there are a number of hypothesis testing methods employed in those tools, choosing an optimal one is thus a challenge for users without enough training in statistics. Therefore, an automatical hypothesis testing method selection function based on intrinsic attributes of meta-omics data will greatly improve user experience.

In this study, we present MetaComp, a graphical software incorporates metagenomics, metatranscriptomics, metaproteomics and metabolomics data by accepting abundance profile matrices (APM) saved as txt or BIOM format [21] and the outputs of BLAST [22], HMMER [23], Kraken [24], MG-RAST [25], MZmine [26] and PhymmBL [27] as input. To reveal the interaction between microbial community and its living condition, a novel quantitative characterization of the effect of environmental factors on microbial community through a nonlinear regression is introduced. MetaComp also provides a series of statistical analysis and the visualization for the comparison of functional, physiological and taxonomic signatures in two-, multi- and two-group sample tests. During two-group comparison, MetaComp is able to automatically select the most appropriate hypothesis testing strategy based upon characteristics of the given data set. Moreover, according to our estimation, the selected hypothesis testing method demonstrates the best performance in comparison among mainly used statistical tools. These novel functions agree with the core concerns of comparative meta-omics in this big data era.

## Implementation

**Fig. 1:**
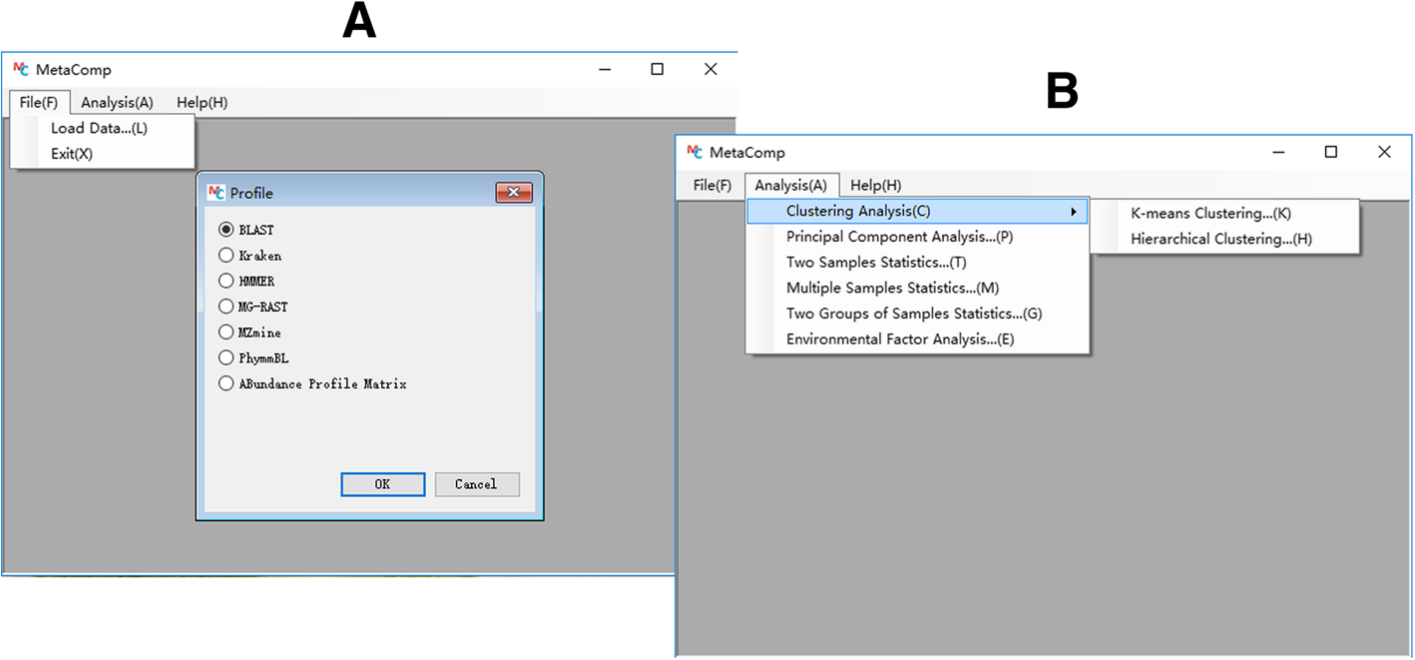
The graphical user interface of MetaComp. (**a**) Drop-down menu *File* for data input. (**b**) Drop-down menu *Analysis* for selecting analysis methods

**Fig. 2:**
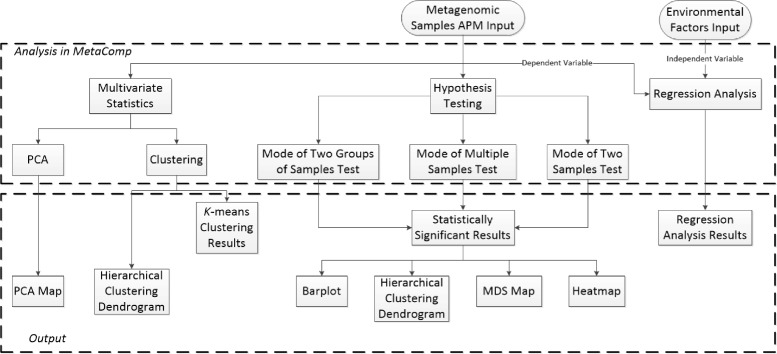
The workflow of MetaComp. The input data of MetaComp includes meta-omics data (for all analyses) and environmental factors input (only for regression analysis). The analysis procedure in MetaComp consist of three independent parts: multivariate statistics (PCA and cluster analysis), statistical hypothesis tests (two-sample test, multi-sample test and two-group sample test) and regression analysis of environmental factors. The outputs are provided in Excel spreadsheet (*k*-means clustering results, statistically significance for each feature and regression analysis results) and visualized in diagrams (PCA map, hierarchical clustering dendrogram, bar plot, MDS map, heat-map)

MetaComp is implemented in C# and R programming languages. The software installer for Windows system, R program and databases of COG, KO and Pfam categories for Linux system and user guide can be found at the website http://cqb.pku.edu.cn/ZhuLab/MetaComp/ or at the GitHub site https://github.com/pzhaipku/MetaComp/. The website of MetaComp provided highlight descriptions, pages about software workflow, convenient download pages, online user guides, detailed demonstration of all application examples with input data and contacts of authors. As illustrated in Fig. 1, MetaComp provides a concise graphical user interface that two drop-down menus are presented: *File* (for data input) and *Analysis* (for analysis method selection). In the following subsections, we first review the preparation of abundance profiles for four types of meta-omics data. Then, based on outputs of these pipelines, we further introduce the various standard input formats for MetaComp. Finally, integrated statistical analysis options and visualization for these analysis are demonstrated. The structure as well as work flow of MetaComp is displayed in Fig. 2.

### Preparation of abundance profiles of meta-omics data

**Fig. 3:**
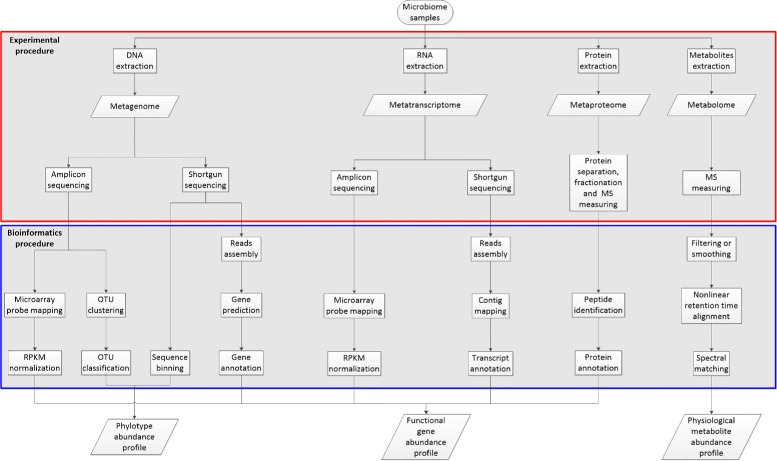
The workflow of preparation for all four types of meta-omics data. Metagenomics, metatranscriptomics, metaproteomics and metabolomics data are preprocessed through experimental procedures such as molecule extraction, sequencing for nucleotides or MS measuring for peptides and metabolites. Then, bioinformatics procedures such as sequence assembly and functional annotation are introduced. Finally, the results of this workflow are functional gene, taxon and physiological metabolite abundance profiles

According to Fig. 3, three types of macromolecules and other metabolites are first extracted from environmental samples separately then sequenced or measured by different techniques. Two major sequencing strategies for DNA and RNA chains are designed in different purposes. Shotgun sequencing is aiming to reflect the global content of metagenome or metatranscriptome by randomly amplifying and sequencing all DNA or RNA sequences, while amplicon sequencing is focused on selected marker genes or 16S rRNA by specifically amplifying primer induced sequences [25, 28]. The metaproteome and metabolome are measured in another routine. Proteins and metabolites are first separated and fractionized by multidimensional liquid chromatography (LC) then measured by tandem mass spectrometer and the final result is mass spectrometry (MS) spectra data [20, 29, 30].

After these experimental processing, the rest procedures for functional gene, taxon and physiological metabolite abundance profiling within a sample are conducted mainly by bioinformatics approaches. There are three major workflows for generalizing functional gene abundance profiles from meta-omics data. The workflow for metagenomics and metatranscriptomics amplicon sequencing data are directly mapped to marker genes through microarray techniques, and after reads per kilobases million (RPKM) normalization or other complicated normalization the gene abundance profiles are obtained.

To extract taxon profile of a metagenomic sample, both 16S rRNA reads and binning results are utilized. The reads of amplicon sequenced 16S rRNA are primarily clustered into operational taxonomic units (OTUs), then each OTU is classified using RDP classifier [31], QIIME [32], Mothur [33] or just BLAST against taxonomic 16S rRNA databases (RDP [34], Greengenes [35], SILVA [36] and NCBI 16S rRNA). Except this procedure, shotgun sequenced genomic reads carry phylogenetic features as well. Based on characterizing nucleotide composition of a read or aligning to reference genomes, a series of approaches denoted as binning are developed. Among these approaches, PhymmBL is the most accurate method, and recently software Kraken achieves a comparable accuracy but consumes less time.

The profiling of shotgun sequencing metageomics data is consist of three steps: reads assembly, gene prediction and gene annotation. DNA reads are first assembled into contigs or scaffolds through IDBA-UD [37], CABOG [38], MAP [39] or InteMAP [40]. After that, MetaGeneMark [41], Glimmer-MG [42] or MetaGUN integrated with MetaTISA [43, 44] are adopted for gene prediction. MetaGeneMark [41] and Glimmer-MG [42] are able to perform a solid detection for known coding genes within metagenomic contigs, while MetaGUN further enables to discover novel genes through domain based searching strategy [43]. At last, by utilizing BLAST, HMMER or MG-RAST to search in ontology databases including COG [45], KO [46], Pfam [47] and SEED [48], the functional profile for metagenomics data is obtained.

Though the processing of shotgun sequenced metatranscriptomics data is consist of three steps as well, the second step of transcriptomic analysis is contig mapping other than gene prediction. After assembled by trinity [49], RNA contigs and scaffolds are simply mapped to reference genomes or Uniprot database [50] utilizing BWA [51] or Bowtie [52] program. The functional profile is obtained in the same way as that for metageomics data.

The LC-MS measured metaproteomics data are profiled in just two steps: peptide identification and protein annotation. As for peptide identification step, MS data are matched with amino acid or nucleotide sequences via search engines such as SEQUEST [53] and Mascot [54]. Then, it shares the same functional annotation step with metagenomic and metatranscriptomic analysis.

The physiological biomarker reflected by metabolomics data are detected in a unique procedure and consist of tandem MS data filtering or smoothing, nonlinear retention time alignment of peaks and spectral matching of the tandem MS data to METLIN [55] and MassBank [56] databases. This pipeline can be realized by MZmine [26] and XCMS [20] tools, resulting in fully annotated MS profiles of metabolites.

### Standard input formats

Though the output file formats of all these mentioned softwares are largely different, they are regarded as standard inputs of MetaComp. The functional abundance profiling are mainly conducted by BLAST and HMMER at the last annotation step, and only a few meta-omics data are offered in tab separated variables form as MG-RAST. For taxon abundance profiling, many OTU clustering programs (e.g. QIIME, Mothur and RDP classifier) employ BIOM format files as output, meanwhile binning programs always offer simply two column hit results. Besides, the output of physiological biomarker detection is always arranged in a tabular format such as MZmine. After loaded, input files are automatically transferred into APM whose rows correspond to features and columns correspond to individual meta-omic samples. Moreover multiple file selection is supported. Here, the features refer to functional gene categories or phylotype categories. The total number of features *i* (*F*_*i*_) observed in metagenomic sample *j* (*S*_*j*_) is represented by *c*_*ij*_ (see Table 2).

**Table 2:** Input data of MetaComp

	*S*_1_	*S*_2_	…	*S*_*n*_
*F*_1_	*c*_11_	*c*_21_	…	*c*_*n*1_
*F*_2_	*c*_12_	*c*_22_	…	*c*_*n*2_
*F*_3_	*c*_13_	*c*_23_	…	*c*_*n*3_
…	…	…	…	…
*F*_*m*_	*c*_1*m*_	*c*_2*m*_	…	*c*_*nm*_

### Statistical analysis options and visualization

We integrated a series of statistical analysis options in MetaComp (see Fig. 2), ranging from descriptive multivariate statistical analyses, hypothesis testing analyses, nonlinear regression analysis of environmental factors and corresponded visualization. Herein, we introduce each statistical analysis option in the following paragraphs.

#### Multivariate statistics

MetaComp employs principal component analysis (PCA) and clustering approaches (e.g. *k*-means clustering and hierarchical clustering) to present an overview of the differences among the given sets of meta-omics samples and highlight main features for each sample. Though it is a descriptive statistical function, these results are indispensable visualizations of meta-omics features. For example, enterotypes is illustrated by PCA figure.

#### Statistical hypothesis tests

Statistical hypothesis tests for comparative meta-omics are provided in MetaComp through three test modes:

- *Mode of two-sample test:* As the amount of meta-omic features is usually huge, we choose *z*-test instead of *t*-test as our default method to assess statistical significant differences between two individual samples. Thus *z*-score for the feature *F*_*i*_ is read as
  1 $$ z_{i} = \left(\frac {c_{i1}}{N_{i1}} + \frac {c_{i2}}{N_{i2}}\right)/\sqrt{P(1-P)\left(\frac {1}{N_{i1}}+\frac {1}{N_{i2}}\right)},  $$
  where $N_{i1}=\sum _{i=1}^{m} c_{i1}$, $N_{i2}=\sum _{i=1}^{m} c_{i2}$ and *P*=(*c*_*i*1_+*c*_*i*2_)/(*N*_*i*1_+*N*_*i*2_). Since *z*-test is not valid if the feature size is insufficient, the prerequisite of *z*-test is $\min (c_{i1}, c_{i2})\leqslant z_{i}^{2}$. When the sample size is small or user demands a more strict hypothesis testing method, MetaComp also offers Fisher’s exact test as an alteration (see the user guide of MetaComp for detailed recommendation).
- *Mode of multi-sample test:* In this mode, pairwise tests between all conceivable pairs of samples are executed by *z*-test. The *p*-value of a specific feature *i* is the minimum of all conceivable *p*-values. Thus we can identify that the selected feature is significantly different in at least one pair of samples.
- *Mode of two-group sample test:* During this test, all samples are classified into two groups. In MetaComp, we provide four statistical hypothesis test methods (*t*-test, paired *t*-test, Mann-Whitney *U* test and Wilcoxon signed-rank test) to assess whether a specific feature is significantly different between two groups of samples. Users can choose a proper method themselves or let MetaComp determine the most suitable test method according to the criterion shown in Table 3.

**Table 3:** Criterion for selecting appropriate test

	Parametric	Non-parametric
Independent	*t*-test	Mann-Whitney *U* test
Correlated	Paired *t*-test	Wilcoxon signed-rank test
  If MetaComp judges that input data follow a Gaussian distribution, parametric hypothesis testing should be introduced. Otherwise when sample size is small or normality assumption is violated, nonparametric hypothesis testing should be conducted. If two groups of samples are consist of matched pairs for resemble units, or one group of units that has been tested twice, it indicates that two groups of samples are correlated. This automatical selection will be helpful for users lacking of adequate statistical training.
  Moreover, odds ratio (OR) test was also implemented to evaluate the relative abundance for each feature as Table 4 demonstrated.

**Table 4:** Contingency table for odd ratio test

	*G*_1_	*G*_2_	Sum
*F*_*j,j*=*i*_	$M_{11}=\sum \limits _{j\in G_{1}}c_{j1}$	$M_{12}=\sum \limits _{j\in G_{2}}c_{j2}$	$n_{1}=\sum \limits _{l=1}^{2}M_{1k}$
*F*_*j,j*≠*i*_	$M_{21}=\sum \limits _{j\notin G_{1}}c_{j1}$	$M_{22}=\sum \limits _{j\notin G_{2}}c_{j2}$	$n_{2}=\sum \limits _{l=1}^{2}M_{2k}$
Sum	$M_{1}=\sum \limits _{j=1}^{2}M_{j1}$	$M_{2}=\sum \limits _{j=1}^{2}M_{j2}$
  Here, *G*_1_ and *G*_2_ is in short for Group 1 and Group 2. *c*_*jk*_ denotes as counts for the *j*-th feature from the *k*-th group samples.
  Considering the possibility of unevenness between two groups, an empirical continuity correction has been introduced to improve the accuracy of the test. Consequently, OR statistic for feature *i* is
  2 $$ \log_{2} {OR(i)} = \log_{2} {\frac {\left(M_{11} + \frac{R}{R+1}\right)\left(M_{22} + \frac{1}{R+1}\right)}{\left(M_{12} + \frac{1}{R+1}\right)\left(M_{21} + \frac{R}{R+1}\right)}}.  $$
  Where *R*=*M*_1_/*M*_2_. According to the formula above, features are categorized as group 1 enrichment (when log2*OR*(*i*)>1) or group 1 scarcity (when log2*OR*(*i*)<1).

#### Multiple test correction

As the typical meta-omics profile consists of hundreds to thousands of features (e.g. Pfam/COG functional profiles), direct application of statistical method described above may probably lead to large numbers of false positives. For example, choosing a threshold of 0.05 will introduce 500 false positives in a profile contains 10000 features. Therefore, two correction methods are implemented in the MetaComp software to solve this problem, including false discovery rate (FDR) as the default option and a stricter option Bonferroni correction.

#### Regression analysis of environmental factors

MetaComp provides a novel function, regression analysis of environmental factors, which means regression analysis of the influence exerted by environmental factors on microbial communities. This original function is implemented by nonlinear regression analysis via the lasso algorithm. MetaComp first normalizes the data of both meta-omics samples and environmental factors. After that, the *i*th environmental factor in *j*th sample (which we shall denote by *x*_*ij*_) is considered as independent variable, and the *j*th frequency of *k*th feature (which we shall denote by *y*_*kj*_) is considered as dependent variable. Therefore, the regression function is:

3 $$ y_{kj}=\sum_{i} {\alpha_{ki}x_{ij}}+\sum_{m,n}^{m\neq n}{\beta_{kmn}x_{mj}\cdot x_{nj}}   $$

where *x*_*mj*_·*x*_*nj*_ means the co-effect of environmental factor *x*_*mj*_ and *x*_*nj*_ to feature *y*_*kj*_. Then, *α*_*ki*_ and *β*_*kmn*_ represent the regression coefficient of the function. For any specific feature, the influence of environmental factors on samples is appraised by coefficient value and correlation value. Moreover, the reliability of the regression coefficient is estimated by *p*-value. Only when all *p*-values meet the prescribed standard, the result of regression would be accepted by MetaComp.

#### Visualization of statistical significance analysis

**Fig. 4:**
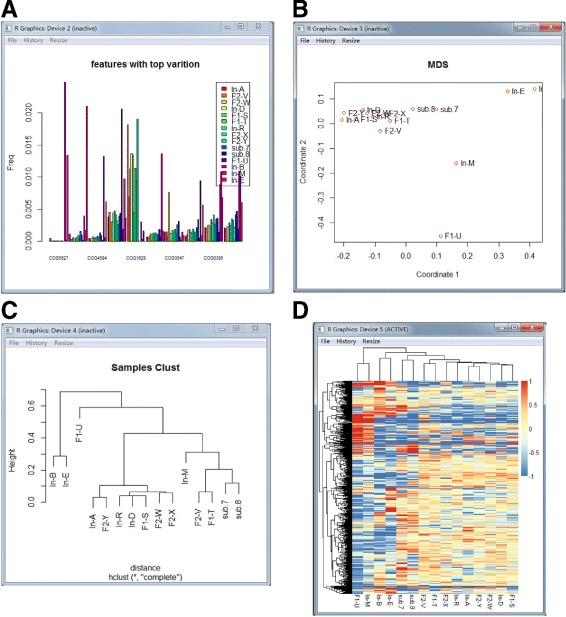
The visualization examples of MetaComp. **a** The bar plot of the top ten significantly different features. **b** The multi-dimensional scaling map of samples. Each point represents an individual sample. **c** The hierarchical clustering dendrogram of given samples. **d** The heat-map of given samples
- *Hierarchical clustering dendrogram and multi-dimensional scaling map:* Hierarchical clustering dendrogram and multi-dimensional scaling map are presented to illustrate the clustering and distance information of meta-omics samples respectively. Features with significant differences (*p*<0.05) are involved in this clustering.
- *Two-dimensional heat-map:* Two-dimensional heat-map is performed to investigate the relative abundance of each feature and the similarity among independent samples.

For the MetaComp software, the visualizations of the hypothesis testing results are displayed in Fig. 4, including:

- *Bar plot:* Bar plot is exhibited for the top 10 significantly different features with their frequencies in each sample.

Moreover, our software enables to save the figures in many formats (e.g..eps,.pdf,.png and.jpeg etc.) that can be used directly for publication.

## Results and discussion

### Analysis process

The analysis workflow of MetaComp can be described as follows (see Fig. 2 for a graphical overview):

- Meta-omics input data are loaded for the further statistical processing through *File* menu. Outputs of BLAST, HMMER, Kraken, MG-RAST, MZmine and PhymmBL, BIOM format and APM saved as txt files are able to load by MetaComp. Additional environmental factors input data are required if users intend to conduct environmental factors analysis on APMs of samples. These environmental factors are also arranged as APMs.
- After loading input data, users should choose an analysis from multivariate statistics, statistical hypothesis tests and environmental factor analysis. The option is made through *Analysis* menu and parameters is set in pop-up dialog boxes.
- The result of analysis is displayed as Excel spreadsheet with corresponding visualization.

### Application in comparison of meta-omic samples

There are four types of meta-omics data characterizing microbiota in different levels but revealing two types of information—static composition of taxon as well as functional gene and dynamic gene expression condition of a microbial community. Metagenomics data including 16S rRNA sequences provide an overview of both phylogenetic and functional gene composition, however metatranscriptomics, metaproteomics and metabolomics data decipher the functional response of a microbiota to various environmental perturbations over spatial and temporal scales. Particularly, metatranscriptome and metaproteome are quite similar and aiming to reflect the fluctuation of functional gene expression, meanwhile metabolome complement with metabolic flux variations of biological pathways via specific physiological biomarkers to unveil the functional gene regulation indirectly. Metagenomics data provide the universe of all possible protein coding genes and metabolic pathways, meanwhile metatranscriptomics, metaproteomics and metabolomics data identify a subsets of active genes and pathways under specific environment. Besides, according to our application of MetaComp on various types of meta-omics data, though these techniques characterize microbiome in different levels and may introduce concentration instead of abundance or frequency, it seems not result in differences on the features of data itself. Hereafter, we demonstrate that the application of MetaComp in meta-omics data presenting in both compositional and dynamical characterizations.

#### Example 1. eight typical environmental metagenomic samples

**Fig. 5:**
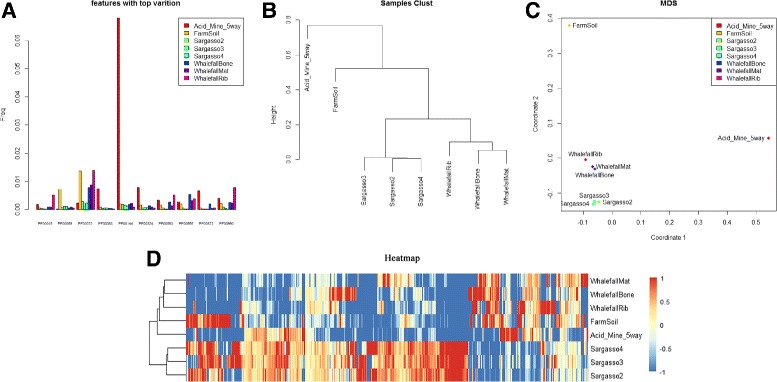
Visualizations of metagenomic samples analysing results. **a** This bar plot displays the top ten significantly different protein families among eight given samples. The frequencies of PF00072, PF00144, PF00872 in eight samples are dramatically fluctuated. **b** Hierarchical clustering dendrogram of eight given samples. **c** Multi-dimensional scaling map of eight given samples. Obviously, three samples from Sargasso Sea as well as three whale fall samples are grouped respectively; Minnesota farm soil and AMD samples are separated from Sargasso Sea samples and whale fall samples in both phylogenetic view and multi-dimensional distance. **d** The heat-map of eight given samples. This figure demonstrates our conclusion mentioned above through the similarity of relative gene abundance among eight samples

Herein we analysed eight typical environmental metagenomic samples, including whale fall, Sargasso Sea, Minnesota farm soil and AMD, which were originally compared by Tringe et al. [57] (input data are listed in Additional file 1: Table S1). The input shotgun sequenced data was annotated by Pfam database. Though amplicon sequenced 16S rRNA data was not included in this example, the processing was all the same as for shotgun sequenced metagenomic data. So that we only focused on comparing shotgun sequenced data in this case. During this analysis, we chose multi-sample test and the results clearly illustrate that the protein family profile of a microbial community is similar to that of other communities when their living environments are highly analogous (illustrated in Fig. 5). According to the detailed analysis results demonstrated in Additional file 2: Table S2, 3456 protein families are significantly different (FDR <0.01) among all given 11,110 compared protein families. These different features are closely related to the living conditions of metagenomic samples. For example, a large amount of *bacteriorhodopsin*-like proteins (e.g. PF01036) are found in all three Sargasso Sea samples, while these proteins are hardly detected in other samples. This protein is involved in obtaining light energy. In addition, since the content of potassium is apparently higher in AMD and soil, the quantity of potassium ion channel protein (e.g. PF03814, PF02705) in AMD and Minnesota farm soil greatly surpasses that in other samples (shown in Table 5).

**Table 5:** Part of whale fall, Acid Mine Drainage, Sargasso Sea, and Minnesota soil metagenomic samples analysis result

	AMD	Soil	S.2^a^	S.3	S.4	W.Bone^b^	W.Mat	W.Rib	*p*-value	*q*-value	Function
PF01036	0	1	344	354	332	0	0	0	1.60e-35	4.67e-34	Bacteriorhodopsin-like protein
PF03814	99	17	3	4	7	0	0	1	5.22e-48	2.92e-46	Ion channel KdpA Potassium-transporting ATPase A subunit
PF02705	0	87	15	30	30	10	0	1	2.27e-56	3.59e-54	APC K trans K ^+^ potassium transporter
PF01077	42	4870	62	51	71	57	45	37	0	0	NIR_SIR Nitrite and sulphite reductase 4Fe-4S domain

^a^S =Sargasso Sea

^b^W=Whale Fall

#### Example 2. Acid Mine Drainage metaproteomic samples

Due to similarity on characterizing dynamics of functional gene expression in a microbiota, it is enough to choose either metatranscriptomic samples or metaproteomic samples to test MetaComp performance. We then take metaproteomic samples of membrane and cytoplasmic proteins from biofilms at B-drift site of Richmond mine as input data for MetaComp. The biofilms were classified into early (labeled as GS0), intermediate (labeled as GS1) and late (labeled as GS2) growth stages. Significantly correlated proteins were identified by significance analysis of microarrays (SAM) or clustered by self-organizing tree algorithm (SOTA) in previous study (see Additional file 3: Table S3 for more details) [10]. Since MetaComp is designed for count data which means no negative variables is allowed as input, we transformed the original relative abundance data exponentially, with the base as 10.

Herein, we conducted two-sample *z*-test for these three samples. The results agree with the previous classification in most cases. For instance, 91.9% of early growth stage, 93.2% of late growth stage and 83.3% of intermediate growth stage expressed genes identified either by SAM or SOTA are also recognized by MetaComp. In addition, the rest proteins cannot provide comparing result due to too low abundance among compared samples.

We further observed that abundance of 65 out of 144 proteins identified previously as early stage expressed demonstrate significantly lower (*p*<0.05) in early growth stage than intermediate stage. Meanwhile, previously identified intermediate stage expressed proteins indicate a *p*-value less than or equal to 4.18×10^−30^. With this *p*-value as threshold, 19 proteins still express significantly larger in intermediate stage than early stage, within which 10 proteins are engaged in environmental sensing procedure, others also correspond with specific cell processing and metabolic processing (see Additional file 4: Table S4 and Additional file 5: Figure S1 to S3 for more details). For example, LeptoII_Cont_10776_GENE_10 annotated as an important heat shock protein—GroEL, is regulated by RNA polymerase subunit *σ* ^32^ during heat stress [58]. LeptoII_Scaffold_8241_GENE_340 annotated as Acetyl-CoA synthetase is also demanded in stationary phase rather than exponential phase to reduce fatty acids generated from membrane lipids [59]. Moreover, flagella synthesis related proteins (LeptoII_Scaffold_8241_GENE_209 annotated as FlgD, LeptoII_Scaffold_8241_GENE_653 annotated as FliD and LeptoII_Scaffold_7904_GENE_5 annotated as FlhA) are classified as intermediate expressed protein by MetaComp. According to the previous results [10], other flagellar proteins are expressed during intermediate and late stages of growth. We further noticed that LeptoII_Scaffold_8241_GENE_209, LeptoII_Scaffold_82 -41_GENE_653 and LeptoII_Scaffold_7904_GENE_5 only take parts in middle procedures of flagella biosynthesis other than from the beginning procedures [60]. Therefore, these genes identified as mainly expressed in intermediate stage by MetaComp is reasonable (these genes are listed in Table 6).

**Table 6:** Part of early and intermediate stage gene analysis result

Protein ID	KO	Early stage	Intermediate stage	*p*-value	Function	Annotation
LeptoII_Cont_10776_GENE_10	K04077	2.38	6.94	4.88e-32	Cellular Processing	Chaperonin GroEL
LeptoII_Scaffold_8241_GENE_340	K01895	1.96	6.35	3.07e-30	Environmental sensing	Acetyl-CoA synthetase
LeptoII_Scaffold_8241_GENE_209	K02389	2.57	6.78	1.72e-30	Environmental sensing	Probable flagellar hook capping protein (FlgD)
LeptoII_Scaffold_8241_GENE_653	K02407	1.32	7.84	1.36e-41	Environmental sensing	Putative flagellar hook-associated protein (FliD)
LeptoII_Scaffold_7904_GENE_5	K02400	0.77	7.63	4.51e-43	Environmental sensing	Probable flagellar biosynthesis protein FlhA

#### Example 3. human fecal metabolomic samples

Since metabolome data indirectly reflect the conditional response of a microbial community, which is distinct with metatranscriptome and metaproteome, it is necessary to examine the performance of MetaComp on this data. We applied MetaComp on metabolomics data of fecal microbiota detected by *Raman* et al. [5]. The original data includes two groups of samples: 30 NAFLD patients for one group and another group with an equal number of healthy volunteers (see Additional file 6: Table S5 for more details). In *Raman’s* study, researchers focused on detecting volatile organic compounds (VOC) which may exert toxic effect to human liver and secreted by human gut microbiota [5]. VOCs were not quantitatively measured but counted by detected or not per individual. Therefore, by gathering this binary counts for both prevalence group and control group, the maximum value for each type of VOC per group is 30.

MetaComp conducted a two-sample *z*-test on NAFLD and control group, and results indicate that 15 out of 220 VOCs are significantly different between two groups (see Additional file 7: Table S6 and Additional file 5: Figure S4 for more details). Furthermore, most VOCs identified as significantly different are included in previous study expect indolizine and acetic acid butyl ester and it may because of lacking of hits (only 5 hits in appeared in healthy control samples) that makes it difficult to be detected in previous study. It is notable that 6 out of 8 VOCs enriched in NAFLD fecal samples are short fatty acid esters. These derivatives of short fatty acids reflect that a relatively high concentration of hexose dietary such as frequently drinking soft drinks with fructose, which is a cause of NAFLD (shown in Table 7) [61].

**Table 7:** Part of nonalcoholic fatty liver disease samples analysis result

Compound	Control	NAFLD^a^	*p*-value
Butanoic acid, propyl ester	1	14	0.0016
Ethyl acetate	0	9	0.0045
Acetic acid, pentyl ester	1	10	0.0112
Propanoic acid, propyl ester	5	18	0.0142
Butanoic acid, 3-methyl-, butyl ester	1	9	0.0183

^a^NAFLD =nonalcoholic fatty liver disease

### Application in regression analysis of environmental factors

#### Example 1. Hawaii Ocean metagenomic samples

**Fig. 6:**
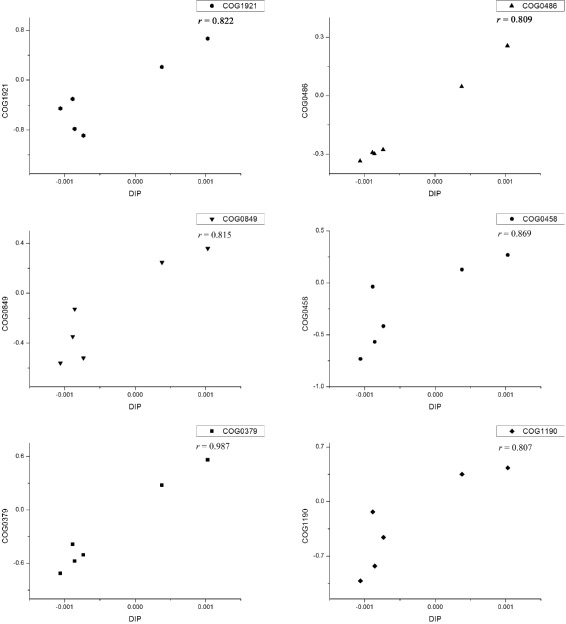
Diagrams of regression. These diagrams exhibit the relationship between DIP and selected functional genes categorized by COG (COG0379, COG0458, COG0486, COG0849, COG1190 and COG1921). It is obviously that the abundance of these genes is linear with the content of DIP

We applied the novel function of MetaComp, regression analysis of environmental factors, on metagenomic samples from Hawaii Ocean [7] (see Additional file 8: Table S7 for more details). The input reads were aligned against COG database [45]. The selected environmental factors are dissolved inorganic phosphate (DIP) and oxygen content (OC) (see Additional file 9: Table S8 for more details). Concluded from the detailed analysis results (see Additional file 10: Table S9 for more details), we discover 102 out of 4,873 COGs which are probably corresponding to the living environment of Hawaii Ocean (*p*<0.1). Moreover, some of the selected COG features are related to the generation and consumption of Adenosine Triphosphate (ATP), which is evidently related to phosphate and oxygen. For instance, COG0378, as a Ni ^2+^-binding GTPase involved in regulation of expression and maturation of urease and hydrogenase, will generate organic phosphorus as well as dehydrogenase. Moreover, this reaction may consume oxygen. It is obvious that this COG is linked to both DIP and OC. Details of these protein families are shown in Table 8. Meanwhile, COGs relevant to the content of DIP (e.g. COG0379, COG0458, COG0486, COG0849, COG1190 and COG1921) are selected by MetaComp (illustrated in Fig. 6).

**Table 8:** Part of relationship between metagenomic samples and environmental factors analysis result

	Coefficient			*p*-value	Correlation	Annotation
	DIP^a^	OC^b^	DIP&OC			
COG0378	0	0	3.303e-06	2.97e-02	9.01e-01	Ni ^2+^-binding GTPase involved in regulation of expression and mature
COG1921	1.157e-03	0	0	8.41e-02	8.22e-01	Selenocysteine synthase [seryl-tRNASer selenium transferase]
COG0318	4.841e-03	0	0	8.97e-02	8.15e-01	Acyl-CoA synthetases (AMP-forming)/AMP-acid ligases II

^a^DIP =dissolved inorganic phosphate

^b^Oxygen=oxygen content

#### Example 2. Acid Mine Drainage metagenomic samples

A total of 40 AMD samples distinct in environmental characteristics were previously collected across Southeast China. Sampling procedure and data processing were described previously [62] (see Additional file 11: Table S10 for more details). The measured environmental factors were dissolved oxygen (DO), total organic carbon (TOC) and SO$_{4}^{2-}$ (see Additional file 12: Table S11 for more details). According to the detailed analysis results (see Additional file 13: Table S12 for more details), we discover 69 out of 142 genes which are presumably related to the living environment (*p*<0.1). Among the selected genes, fumarate and nitrate reductase (fnr) gene is related to the respiratory chain of bacteria and the reaction of it requires a mass of sulfur. Therefore the abundance of fnr is apparently linked to DO and SO$_{4}^{2-}$. Furthermore, ammonia monooxygenase A (amoA) is an enzyme, which catalyses nitration reaction. This reaction may consume organic carbon and oxygen. It is manifestly related to the content of TOC and DO.

### Compared with other tools in differentially abundant features detection

Variations embedded in meta-omics are always difficult to recognize when the hit number of a feature is slightly different from one group of samples to another but evidently fluctuated among samples of the same group. Since that, to evaluate the differentially abundant features detection ability of current comparative meta-omics methods, we simulated two groups of count data from twin gut samples [2], of which 1,649,149 hits for 1000 COG features was annotated through BLAST against COG database, to reserve the complexity of true data. Then we take a previous evaluation study for reference [63], samples are first amplified with fold change *q*=1.25 and resampled into two equal sized groups of samples through randomly sampling without replacement. After that, 10% of COGs from the second group were chosen by chance and downsampled according to binomial distribution. Herein, we denoted $x_{ij}^{\prime }$ as hits of the *j*-th feature from the *i*-th sample and it followed binomial distribution *B*(*x*_*ij*_,*p*), where *x*_*ij*_ was hit number after resampling and *p*=1/*q* to control the alteration between two groups. Therefore, we obtained two groups of samples with hits of 1,800,000 and 1,744,981 in total, respectively. The dataset generated from resampling is demonstrated in Additional file 14: Table S13.

Since *t*-test (employed by Fantom, STAMP, Metastats, XCMS and MetaComp in two-group sample test), paired *t*-test (employed by XCMS and MetaComp in two-group sample test for correlated samples), non-parametric *t*-test (employed by STAMP in two-group sample test), Wilcoxon Mann-Whitney *U* test (employed by XCMS and MetaComp in two-group sample test for independent samples) and Wilcoxon signed-rank test (employed by XCMS and MetaComp in two-group sample test for correlated samples) were mainly employed hypothesis testing methods, we took these methods for camparison. The result is listed in Table 9, and the detailed significance detection are presented in Additional file 15: Table S14. Under the threshold of FDR <0.05, it is obvious that MetaComp automatically adopted Wilcoxon signed-rank test performed the best over other methods with the highest sensitivity (100.0*%*) and a decent specificity (99.9*%*). Here, sensitivity (SN) and specificity (SP) were calculated with true positive (TP), true negative (TN), false positive (FP) and false negative (FN) as:

4 $$ SN = \frac{TP}{TP+FN},\quad SP = \frac{TN}{TN+FP}.  $$

**Table 9:** Comparison of two-group sample test methods (FDR <0.05)

Hypothesis test	True positive	False negative	False positive	True negative	Sensitivity	Specificity
Non-parametric t-test	65	35	0	900	65.0%	100.0%
Paired t-test	100	0	386	524	100.0%	57.6%
t-test	65	35	0	900	65.0%	100.0%
Wilcoxon rank-sum test	44	56	0	900	44.0%	100.0%
Wilcoxon signed-rank test^a^	100	0	1	899	100.0%	99.9%

^a^This method is automatically selected by MetaComp

**Fig. 7:**
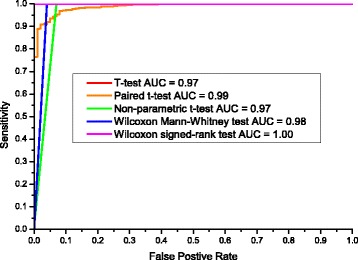
ROC curve for all five methods. ROC performance of five methods in significant feature detection

We further plotted the Receiver operating characteristic (ROC) curve (shown in Fig. 7) to demonstrate the performance of all five hypothesis testing methods as well. This analysis indicated that area under ROC curve (AUC) was almost 1.0 and confirmed automatical selection (other than recommended by XCMS) of MetaComp was the most appropriate one.

## Conclusion

Compared with the previously developed tools, MetaComp takes advantages in three fields.

- Our software is universally applied in all types of meta-omics data including metagenomics, metatranscriptomics, metaproteomics and meta-bolomics data.
- It can be utilized in revealing the relationship between environmental factors and meta-omic samples directly through a nonlinear regression analysis.
- MetaComp is capable of automatically selecting the proper statistical method in two-group sample test thus improving experience for users that are not expertises of statistics.

MetaComp, as comprehensive analysis software for comparative meta-omics, takes advantages in incorporation of all types of meta-omics data, nonlinear regression analysis on environmental factors and automatical selection of suitable tests in two-group sample situation. These improvements meet the major demands in big data era of all types of meta-omics data. Moreover, according to our evaluation, MetaComp outperforms other methods by the automatically selected hypothesis testing method in detection of differentially abundant features. In brief, MetaComp is an integrative comparative meta-oimcs software designed for uncovering biological significant differences and providing visualization of these results for biologists. Moreover, it will throw light upon future comparative meta-omics studies on the complicated relationship between microbes and their living environments.

## Availability and requirements

**Project name:** MetaComp.**Project home page:** Homepage: http://cqb.pku.edu.cn/ZhuLab/MetaComp/GitHub page: https://github.com/pzhaipku/MetaComp/ **Operating system(s):** Linux and Windows 7, 8 and 10.**Programming language:** C# & R**Other requirements:** R 3.1.3 or higher.**Any restrictions to use by non-academics:** none.

## Additional files

Additional file 1 **Table S1**. The input functional gene APM data of eight typical environmental metagenomic samples. (TXT 291 kb)

Additional file 2 **Table S2**. The detailed analysis results of eight typical environmental metagenomic samples. (XLS 2109 kb)

Additional file 3 **Table S3**. The input functional gene APM data of Acid Mine Drainage metaproteomic samples. (XLS 83kb)

Additional file 4 **Table S4**. The detailed analysis results of Acid Mine Drainage metaproteomic samples. (XLS 132 kb)

Additional file 5 **Figure S1** to **Figure S4**. Visualized analysis results for Acid Mine Drainage metaproteomic samples and human fecal metabolomic samples. (PDF 204 kb)

Additional file 6 **Table S5**. The input metabolite APM data of human fecal metabolomic samples from healthy control people and NAFLD patients. (XLS 41kb)

Additional file 7 **Table S6**. The detailed analysis results of human fecal metabolomic samples from healthy control people and NAFLD patients. (XLS 51kb)

Additional file 8 **Table S7**. The input functional gene APM data of Hawaii Ocean metagenomic samples. (TXT 72kb)

Additional file 9 **Table S8**. The input vector of environmental factors related with Hawaii Ocean metagenomic samples. (TXT 1 kb)

Additional file 10 **Table S9**. The detailed analysis results of Hawaii Ocean metagenomic samples. (XLS 50 kb)

Additional file 11 **Table S10**. The input functional gene APM data of Acid Mine Drainage metagenomic samples. (TXT 62 kb)

Additional file 12 **Table S11**. The input vector of environmental factors related with Acid Mine Drainage metagenomic samples. (TXT 1 kb)

Additional file 13 **Table S12**. The detailed analysis results of Acid Mine Drainage metagenomic samples. (XLS 43 kb)

Additional file 14 **Table S13**. The original, resampled and downsampled datasets of twin gut data for comparison of significance detection. (XLS 622 kb)

Additional file 15 **Table S14**. The hypothesis testing results of t-test, paired t-test, non-parametric t-test, Wilcoxon rank-sum test and Wilcoxon signed-rank test. (XLS 168 kb)

## Abbreviations

AMD: Acid mine drainage
amoA: Ammonia monooxygenase A
APM: Abundance profile matrix
ATP: Adenosine triphosphate
AUC: Area under ROC curve
DIP: Dissolved inorganic phosphate
DO: Dissolved oxygen
FDR: False discovery rate
fnr: Fumarate and nitrate reductase
FN: False negative
FP: False positive
IBS: Irritable bowel syndrome
LC: Liquid chromatography
MS: Mass spectrometry
NAFLD: Non-alcoholic fatty liver disease
OC: Oxygen content
OR: Odds ratio
OTU: Operational taxonomic units
PCA: Principal component analysis
ROC: Receiver operating characteristic curve
RPKM: Reads per kilobases million
SAM: Significance analysis of microarrays
SN: Sensitivity
SOTA: Self-organizing tree algorithm
SP: Specificity
TN: True negative
TOC: Total organic carbon
TP: True positive
VOC: Volate organic compounds
