# Supplementary material for: MetaComp: comprehensive analysis software for comparative meta-omics including comparative metagenomics
Source: BMC Bioinformatics. 2017 Oct 2;18:434. doi: 10.1186/s12859-017-1849-8 (PMC5625784; doi:10.1186/s12859-017-1849-8)
Supplement: Supplementary file 5 — Figure S1 to Figure S4. Visualized analysis results for Acid Mine Drainage metaproteomic samples and human fecal metabolomic samples. (PDF 204 kb) [file 12859_2017_1849_MOESM5_ESM.pdf]

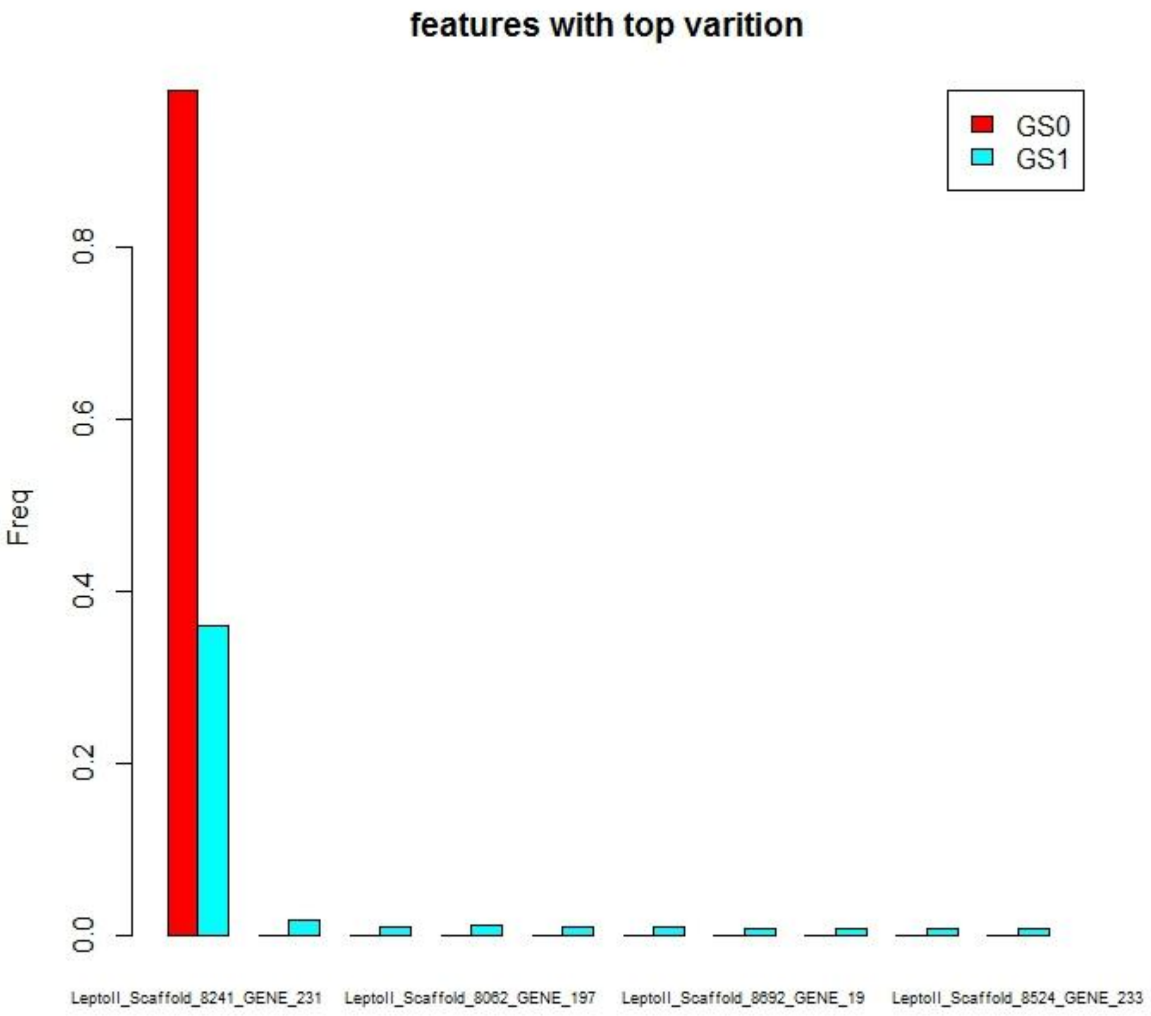

Figure S1: This bar plot displays the top ten significantly different proteins between early (GS0) and intermediate (GS1) growth stages.

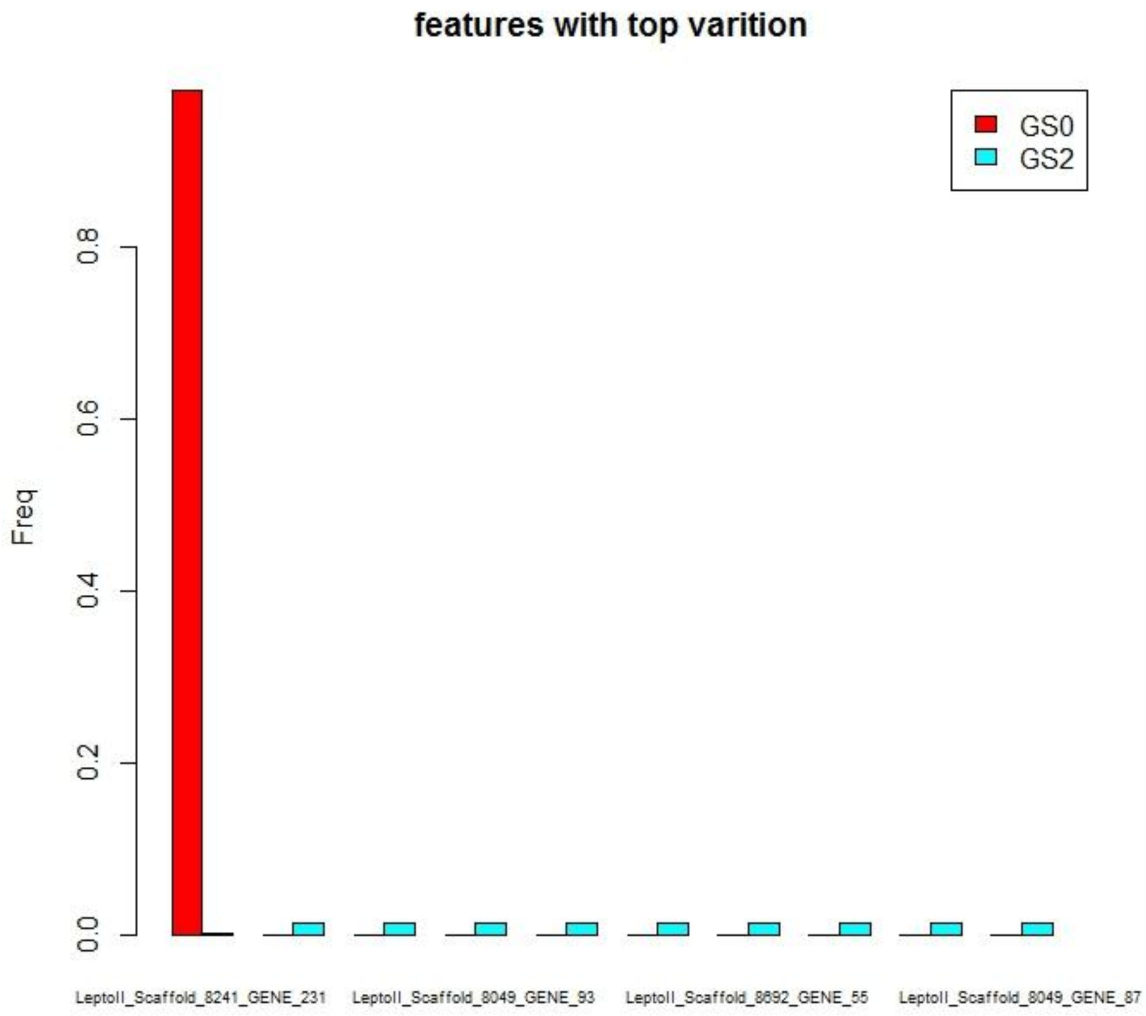

Figure S2: This bar plot displays the top ten significantly different proteins between early (GS0) and late (GS2) growth stages.

.

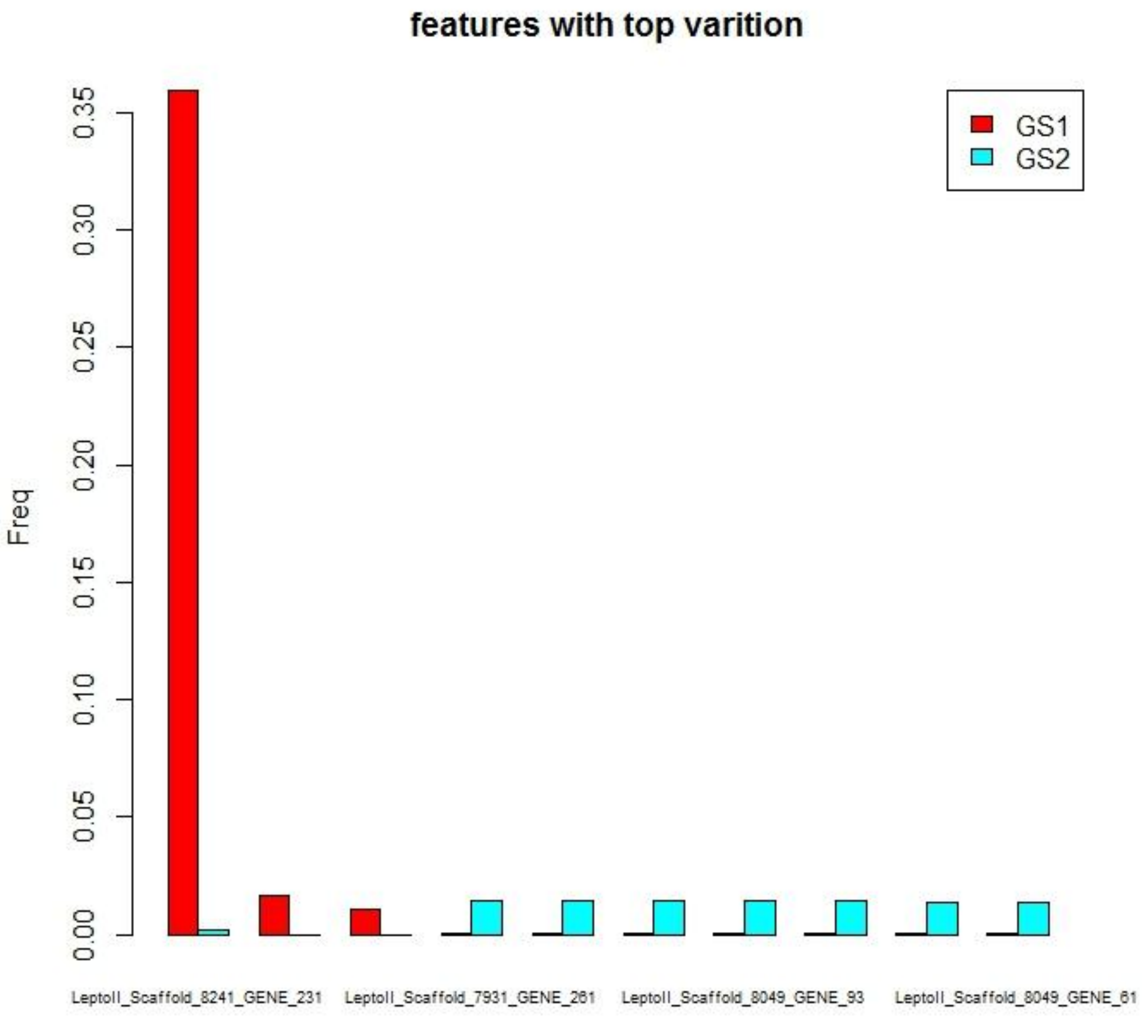

Figure S3: This bar plot displays the top ten significantly different proteins between intermediate (GS1) and late (GS2) growth stages.

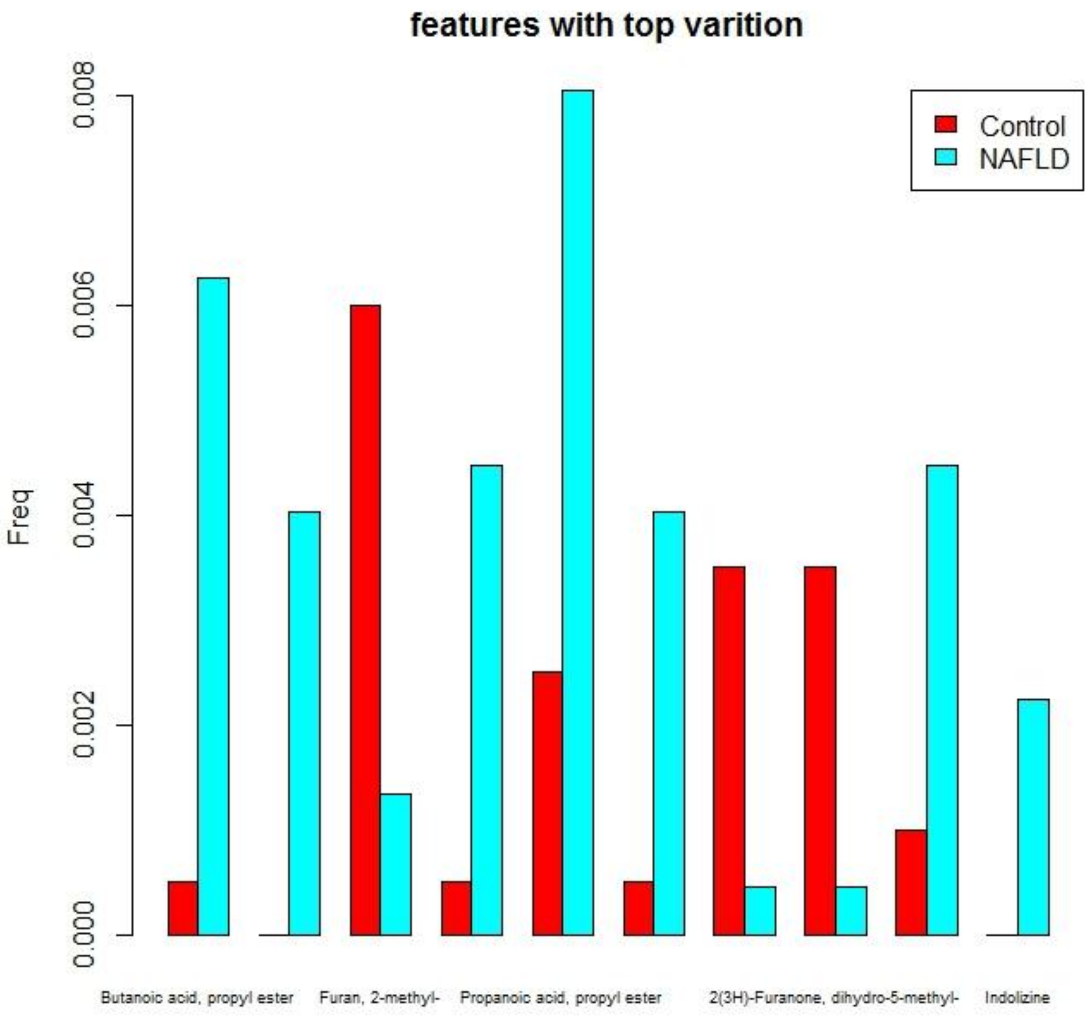

Figure S4: This bar plot displays the top ten significantly different VOC between control and NAFLD group.
